# Discrepancy of eye injuries in mechanism, clinical features, and vision prognosis by different causative sports

**DOI:** 10.3389/fpubh.2023.1182647

**Published:** 2023-10-18

**Authors:** Ying Zhang, Hongzhen Jia, Xin Kang, Qinghua Yang, Jun Ying, Qiong Wu, Zhong Zheng, Hongtao Zhang

**Affiliations:** ^1^Senior Department of Ophthalmology, The Third Medical Center of Chinese PLA General Hospital, Beijing, China; ^2^Department of Ophthalmology of the Sixth Medical Center Stationed by the Senior Department of Ophthalmology of the Third Medical Center of Chinese PLA General Hospital, Beijing, China; ^3^Department of Ophthalmology, The First Medical Center of Chinese PLA General Hospital, Beijing, China; ^4^Information Management Department, Chinese PLA General Hospital, Beijing, China; ^5^Medical Security Center, Chinese PLA General Hospital, Beijing, China; ^6^Information Center, Logistics Support Department, Central Military Commission, Beijing, China

**Keywords:** mechanical eye injury, sports, epidemiology, clinical characteristics, visual acuity, hyphema

## Abstract

**Objective:**

To investigate the epidemiological and clinical characteristics of sports-related eye injuries in China, as well as how they differ depending on the sport or other specific factor that caused them.

**Methods:**

Consecutive medical records from 2015 to 2019 of sports-related eye injuries from a standardized database in nine tertiary referral hospitals in China were retrospectively reviewed and analyzed.

**Results:**

A total of 377 eyes in 376 inpatients (mean age, 22.5 ± 7.3 years; men:women 15.4:1) were included. Soccer (46.8%), basketball (27.1%), and badminton (16.8%) were the top three sports that caused injury. Ball strikes (74.7%), physical collision (13.8%), and racket/equipment beating (9.0%) were the common specific causes of injury. Blunt force injuries (95.8%) and close globe injuries (95.1%) accounted for the majority of injuries. Open globe injuries occurred more in basketball (8.3%) than in other sports, mainly due to physical collision (12.8%) and racket/equipment beating (11.8%). Basketball (13.4%) or physical collision (21.3%) caused Zone I injuries more frequently than other sports. Soccer (60.5%) and basketball (54.6%) caused more injuries to the posterior segment of the eyeball than other sports, mainly due to ball strikes (96.6%). Badminton (69.8%) and racket beating (61.8%) caused more Zone II globe injuries than other sports. In badminton, the percentage of hyphema (85.7%), the most typical symptom of eye damage, and ultimate visual acuity (VA) ≥20/40 (88.9%) was the greatest. A final low vision score of (≤4/200) was observed in 10.6% of all participants, including three participants who had an eye removed due to rupturing. The final VA was positively correlated with the presenting VA (*r* = 0.421).

**Conclusion:**

Sports can lead to high proportions of ocular contusion injury and low vision. VA prognosis is closely related to initial VA following ocular sports trauma, which is directly determined by the causative sports and/or the specific causes. Effective eye protection is imperative to avoid or reduce visual impairments of sports participants.

## 1. Introduction

The eyeball, a fragile and delicate visual organ, is vulnerable to serious damage by trauma, resulting in irreversible blindness and/or disability. About 7.5%–27.3% of eye injuries are sports-related (1–5), which is the leading cause of monocular blindness in the United States (6). An estimated 600,000 ocular sports injuries occur in the United States each year and 13,500 of them result in permanent vision loss (7). In India alone, bilateral ocular trauma occurs in 17.7% of sports-related injuries (4). Ocular sports injuries are, therefore, a worldwide public health concern.

Nearly 90% of ocular injuries from sports are preventable (7). Given its preventability, clinical research can be used to collect data on the incidence and severity of eye injuries in sports events to review the risk of these injuries. Further studies on mechanisms involved in eye injuries can help to develop more effective clinical treatments and to formulate and adjust relevant protective devices, standards, rules and policies, which can prevent visual disability in sports participants as much as possible, thereby gradually reduce the related adverse effects, especially the quality of life among these sports persons and their families (8). Although the popularity and performance of different sports vary in different parts of the world, the basic mechanisms of eye injury caused by certain kinds of sports are similar and can be explored.

So far, China lacks a monitoring and reporting system for sports-related eye injuries and an effective procedure to obtain relevant official data on such injuries. However, it is possible to ascertain the potential of eye injuries in various specific sports and follow up on the results of an intervention through limited, specifically-designed studies. Although there are reports about professional athletes (9) and a specific population (college students whose data was obtained from a questionnaire) (10), as well as reports on certain sports (11), no relevant studies have reported on the overall situation of sports-related eye injuries in the general population in China. This study compiled data on sports-induced eye injuries from multiple centers in China over a 5-year period. Data on specific sports or causes and detailed symptoms of injured eyes and the correlation between them, as well as the influence of specific sports or causes of injuries, are also reported and analyzed comprehensively. The goal of this study is to help protect sports participants from eye injuries as much as possible and improve the effectiveness of relevant treatments and/or preventive measures.

## 2. Materials and methods

In this retrospective case series study, consecutive medical records of all patients with sports-related eye injuries admitted to nine tertiary referral hospitals in China from January 1, 2015 to December 31, 2019 were extracted from a standardized database and reviewed. A total of 377 eyes of 376 inpatients with eye injuries from sports were eligible for analysis.

### 2.1. Study design

Each case was recorded using a standardized preformulated data sheet, and the records were kept in a computerized eye injury database. Detailed information regarding each injured eye following the sports activity, described in the chief complaint and present history, was collected from electronic hospital records, which included patient information (including age and sex); a thorough history (such as related sports items, specific causes of eye injury, timing and nature of injury); clinical presentation, including initial vision following sports injury (presenting vision); and treatments and outcomes, including final vision at discharge or end of follow-up. Findings during surgery provided valid evidence. A completed ophthalmological examination was performed, including slit-lamp examination, ophthalmoscopy, tonometry, ultrasonography, computed tomography, and any other relevant investigations. In this study, all visual acuity (VA) results were either best-corrected or pinhole VA was used. Cases with missing clinical data were excluded, and all included data were cross-checked for errors.

### 2.2. Definitions

Classification and definition of mechanical eye injuries (MEI) in this study was based on the Birmingham Eye Trauma Terminology (12). Open globe injury (OGI) indicates a full-thickness wound of the eyeball and includes rupture (caused by a blunt object, ‘inside out’ mechanism), penetration (caused by a sharp instrument, “outside in” mechanism), intraocular foreign body, and perforation; the latter two are not included in the study data. Close globe injury (CGI) includes contusion (by blunt force, but without global rupture), lamellar laceration (partial thickness wound of the eyeball), and superficial foreign body; the last one is not included in our data.

Globe injury zone was identified in accordance with the Ocular Trauma Classification System (13). The definition of zones in OGI (location of a full-thickness wound of the globe wall) is as follows: I, within the cornea and the limbus; II, scleral area within 5 mm posterior to the corneoscleral limbus; and III, extending 5 mm beyond the limbus. The definition of zone in CGI (involved eye tissues) is as follows: I, ocular surface (limited to the bulbar conjunctiva, sclera and cornea); II, anterior segment structures and pars plana ciliary; and III, intraocular structures behind the posterior capsule interface of the lens.

Chronic hypotony was defined as an intraocular pressure < 8 mmHg at follow-up of at least 6 months. Poor VA referred to a VA ≤4/200 (including the removed eye).

### 2.3. Statistical analyses

Statistical analysis was performed using SPSS version 26.0 (SPSS Inc., Chicago, IL, United States). Three cases with unknown sports items were not included in the group calculation. Categorical variables were analyzed using the chi-square test or Fisher’s exact test when the proportion of cells with theoretical frequency less than 5 exceeded 20%; rank sum test was used for rank data (VA grading). Continuous variables were evaluated for normality, and means were compared using a two-tailed *t*-test. Snellen VA was converted to fractional VA, and Spearman’s rank correlation analysis was performed between initial VA and final VA. A *p* < 0.05 was considered statistically significant for all tests.

## 3. Results

### 3.1. Demographics characteristics

This study analyzed data on 376 patients with 377 eyes injured during sports (binocular injuries, 0.3%; left–right ratio, 1:1.2). There were 22 (5.9%) patients who were highly myopic. The men to women ratio was 15.4:1. The age range was 7 to 53 years (mean: 22.5 ± 7.3 years; median: 21 years).

### 3.2. Eye injuries in sports events and the different specific causes

Ball sports were the leading causes of sports-related eye injuries, followed by racket sports. The top three sports with the highest rates of injuries were soccer, basketball, and badminton, in the respective sequence. Direct ball strikes to the eye were the most common cause of ocular trauma in ball sports (281/376, 74.7%), followed by physical collision (52/376, 13.8%), and a hit by a racket/sports equipment (34/376, 9.0%). Among ball sports, ball strikes accounted for the majority of the causes of injuries in soccer (170/176, 96.6%) and accounted for 56.86% (58/102; *p* < 0.001) of the injuries in basketball. Ocular injuries due to physical collision were more common in basketball (43/102, 42.2%) than in soccer (6/176, 3.4%; *p* < 0.001; Table 1).

**Table 1:** Eye injury counts and rates by different sport events and specific causes.*

Sport events	No. (%)	Specific causes no. (%)			
		Ball strikes	Racket/equipment beating	Physical collision	Tumble/crush injury
Ball Games	279 (74.2)	229 (60.9)	–	49 (13.0)	1 (0.3)
Soccer	176 (46.8)	170 (45.2)	–	6^a^ (1.6)	0
Basketball	102 (27.1)	58 (14.4)	–	43^b^ (11.4)	1 (0.3)
Volleyball	1 (0.3)	1 (0.3)	–	0	0
Racket Games	75 (19.9)	52 (13.8)	23 (6.1)	0	0
Badminton	63 (16.7)	42 (11.1)	21 (5.6)	0	0
Tennis	6 (1.6)	6 (1.6)	0	0	0
Table tennis	4 (1.0)	2 (0.5)	2 (0.5)	0	0
Baseball	1 (0.3)	1 (0.3)	0	0	0
Squash racket ball	1 (0.3)	1 (0.3)	0	0	0
Others	19 (5.1)	–	11 (3.0)	3 (0.8)	5 (1.3)
Power twist	6 (1.6)	–	6 (1.6)	0	0
Fishing	3 (0.8)	–	3^c^ (0.8)	0	0
Parallel bars	2 (0.5)	–	1 (0.3)	0	1 (0.3)
Skating	2 (0.5)	–	–	1 (0.3)	1 (0.3)
Swimming	2 (0.5)	–	–	2^d^ (0.5)	0
Diving	1 (0.3)	–	–	0	1 (0.3)
Rope skipping	1 (0.3)	–	1 (0.3)	0	0
Running	1 (0.3)	–	–	0	1 (0.3)
Riding cycling	1 (0.3)	–	–	0	1 (0.3)
Unknown	3 (0.8)	–	–	–	–

*376 patients in total; percentages were calculated using this as the denominator. A case of binocular injuries caused by ball strikes from soccer.

a 1 eye stabbed by shoes nail and 1 eye stabbed by broken glasses.

b 18 eyes stabbed by fingers and 2 eye stabbed by broken glasses.

c 1 eye beaten by hook and 2 by fishing pendant.

d Eye injury from the swimming goggles beaten by hands.

### 3.3. Types of mechanical eye injuries

A total of 371 eyeballs (98. 4%) were injured, of which CGI accounted for the majority (353/371, 95.1%) and OGI for residual 4.9% (18/371). Contusions predominated in CGI (349/353, 98.9%), and the remaining were laminar lacerations (4/353, 1.1%); ruptures predominated in OGI (12/18, 66.7%), and the remaining were penetrating eye injuries (6/18, 33.3%; Table 2).

**Table 2:** Types of ocular trauma in different sports events and specific causes of 377 injured eyes.

Eye injuries	Close globe injury (*n* = 353)		Open globe injury (*n* = 18)		Eyelid injury (*n* = 42)	Orbital fracture (*n* = 5)
Sports items specific causes	Contusion (*n* = 349)	Lamellar laceration (*n* = 4)	Rupture (*n* = 12)	Penetrating (*n* = 6)		
***Soccer** (n = 177)*	173	*2*	*0*	*2*	*10*	*1*
Ball strikes (*n* = 171)	169	2	0	0	9	0
Physical collision (*n* = 6)	4	0	0	2^a^	1	1
***Basketball** (n = 102)*	*87*	*2*	*6*	*2*	*13*	*1*
Ball strikes (*n* = 58)	52	1	2	2^b^	4	0
Physical collision (*n* = 43)	35	1	3	0	8	0
Tumble injury (*n* = 1)	0	0	1	0	1	1
***Badminton** (n = 63)*	*60*	*0*	*3*	*0*	*6*	*0*
Ball strikes (*n* = 42)	41	0	1^c^	0	2	0
Racket beating (*n* = 21)	19	0	2	0	4	0
***Others** (n = 32)*	*27*	*0*	*3*	*1*	*12*	3
Ball strikes (*n* = 11)	10	0	1^d^	0	2	0
Racket/equipment beating (*n* = 13)	11	0	1^e^	1^f^	4	0
Physical collision (*n* = 3)	1	0	1^g^	0	2	1
Tumble/crush Injury (*n* = 5)	5	0	0	0	4	2
***Unknown** (n = 3)*	*2*	*0*	*0*	1^b^	1	*0*

a 1 eye stabbed by shoes nail and 1 eye stabbed by broken glasses.

b Eyes stabbed by broken glasses.

c The eye with history of penetrating keratoplasty surgery.

d 1 eye beaten by baseball.

e 1 eye beaten by fishing pendant.

f 1 eye stabbed by the broken glasses beaten by bat of table tennis.

g 1 eye hit by other’s head while skating.

**Figure 1:**
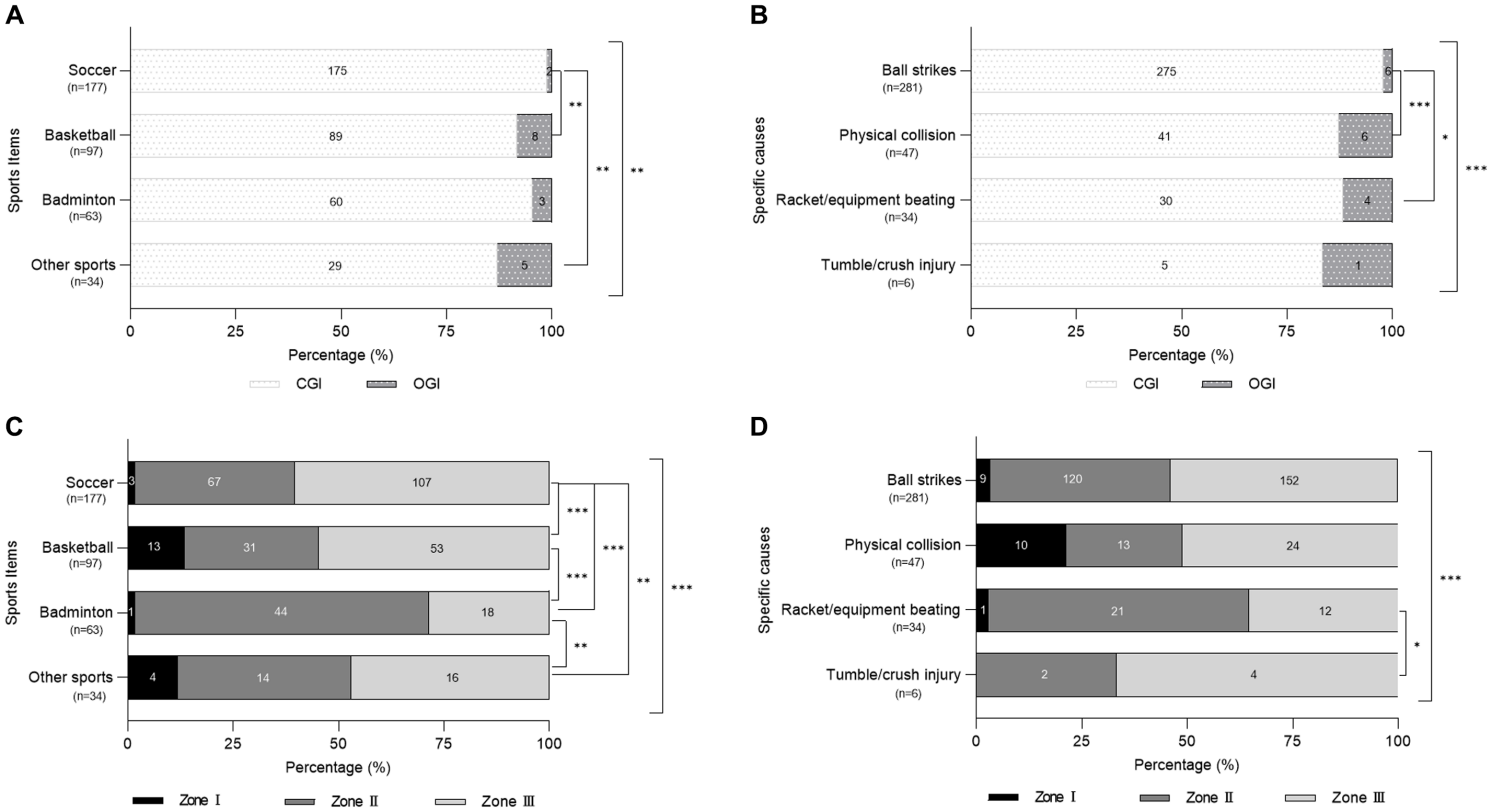
The composition distributions of CGI and OGI **(A,B)** and zones **(C,D)** in mechanical eye injury resulted from different sports items **(A,C)** and specific causes **(B,D)**. 371 eyes with MEI in total; excluding 3 eyes related with unknown specific sports in figure **(D)**. Asterisks indicate significantly different composition proportions (* *p* < 0.05, ** *p* < 0.01, *** *p* < 0.001). CGI, close globe injury; OGI, open globe injury.

The composition of mechanical eye injuries (MEI; CGI vs. OGI) differed in different sports (*p* = 0.001) and with specific causes (*p* = 0.001). Of these, a significantly high proportion of CGIs were observed in soccer (175/177, 98.9%) than in basketball (89/97, 91.8%; *p* = 0.005) and other sports (27/31, 87.1%; *p* = 0.005). Accordingly, OGI occurred more frequently in basketball (8/97, 8.3%) and other sports (4/31, 12.9%) than in soccer (2/177, 1.1%; *p* = 0.005; Figure 1A). Regarding specific causes, ball strikes caused a higher proportion of CGI (275/281, 97.9%) than that caused by physical collision (41/47, 87.2%; *p* < 0.001) and racket/equipment beating (30/34, 88.2%; *p* = 0.015). The proportion of OGI due to ball strikes (6/281, 2.1%) was significantly lower than that due to physical collision (6/47, 12.8%; *p* < 0.001) and racket/equipment beating (4/34, 11.8%; *p* = 0.015; Figure 1B).

### 3.4. Zones of mechanical eye injuries

Zone distributions of MEI among different sports and specific causes varied significantly (*p* < 0.001; Figures 1C,D). The proportion of each zone of MEI in different sports varied (*p* < 0.001). Zone I injury was caused more by basketball (13/97, 13.4%) and other sports (4/34, 11.8%) than by soccer (3/177, 1.7%) and badminton (1/63, 1.6%; *p* < 0.05). Badminton (44/63, 69.8%) caused the most MEI in Zone II (soccer: 67/177, 37.9%, *p* < 0.001; basketball: 31/97, 32.0%, *p* < 0.001; and other sports: 14/34, 41.2%, *p* = 0.006). Soccer (107/177, 60.5%) and basketball (53/97, 54.6%) caused more injuries in Zone III than those caused by badminton (18/63, 28.6%; both *p* < 0.001; Figure 1C).

The proportion of different specific causes of MEI varied in Zone I (*p* < 0.001) and Zone II (*p* = 0.019). Physical collision (10/47, 21.3%) caused more Zone I injuries than that caused by ball strikes (9/281, 3.2%; *p* < 0.001) and racket/equipment beating (1/34, 2.9%; *p* = 0.021). Racket/equipment beating (21/34, 61.8%) caused more Zone II injuries than that caused by ball strikes (120/281, 42.7%; *p* = 0.035) and physical collision (13/47, 27.7%; *p* = 0.002; Figure 1D).

### 3.5. Ocular trauma findings

In ocular sports trauma, hyphema (52.8%) was the most common clinical symptom, with varied proportions in different sports and was highest in the badminton group (54/63, 85.7%; *p* < 0.01). Following vitreous hemorrhage (15.4%), retinal breaks (13.3%) was the second most common clinical sign in posterior segment injuries, with significantly higher proportions in soccer (27/177, 15.3%) and basketball (19/102,18.6%) than in badminton (2/63, 3.2%; *p* = 0.012 and *p* = 0.003, respectively). Traumatic glaucoma (12.7%) was the second most common sign in anterior segment injuries and was significantly higher in badminton (16/63, 25.4%) than in soccer (20/177, 11.3%; *p* = 0.009) and basketball (7/102, 6.9%; *p* = 0.001). In addition, traumatic cataract (6.4%) and traumatic optic neuropathy (6.1%) were other common globe injuries.

### 3.6. Treatments

Surgery was performed in 21.5% of the injured eyes, including intraocular surgery in 13.3% and vitrectomy in 6.4% (Table 3); 36.0% (18/50) underwent intraocular surgeries two or three times. Lens surgery was performed in 5.3% of the eyes (20/377) with intraocular lenses implanted in 2.4% (9/377); glaucoma filtration and ciliary body suturing were performed in 0.5% (2/377) each; scleral buckling performed in 5.8% (22/377), of which vitrectomy was additionally performed in 2.4% (9/377); and keratoplasty, optic nerve sheath decompression, and orbital fracture repair were performed in a total of 0.3% (1/377 for each procedure). Finally, three eyes (0.8%) were removed, including two enucleated eyes within 24 h after injury and 1 eviscerated eye within half a month of injury.

**Table 3:** Surgical treatments in different sports and specific causes of 377 injured eyes no. (%).*

Activity (*n* = 377)	Eye surgery *n* = 81 (21.49)	Intraocular surgery *n* = 50 (13.26)	Vitrectomy *n* = 24 (6.37)	Eye removal *n* = 3^†^ (0.80)
Sports items				
Soccer (*n* = 177)	20 (11.3)	13 (7.3)	8 (4.5)	0
Basketball (*n* = 102)	36 (35.3)	17 (16.7)	10 (9.8)	2 (2.0)
Badminton (*n* = 63)	11 (17.5)	10 (15.9)	3 (4.8)	0
Others (*n* = 35)	14 (40.0)	10 (28.6)	3 (8.6)	1 (2.9)
Specific causes				
Ball strikes (*n* = 282)	45 (16.0)	30 (10.6)	18 (6.4)	0
Physical collision (*n* = 52)	23 (44.2)	8 (15.4)	3 (5.8)	2 (3.9)
Racket/equipment beating (*n* = 34)	8 (23.5)	8 (23.5)	3 (8.8)	0
Tumble/crush injury (*n* = 6)	2 (33.3)	2 (33.3)	0	1 (16.7)
Unknown (*n* = 3)	3 (100.0)	2 (66.7)	0	0

*Percentages were calculated using the total number of each row as the denominator. ^†^3 eyes of severe rupture were removed. Two in basketball (1 injured by a fall, 1 hit by the upper limb); and one hit by other’s head while skating.

The proportions of surgeries related to various sports and specific causes are shown in Table 3. Intraocular surgery for eye injuries were significantly different among sports and various other causes (*p* = 0.003 and *p* = 0.009, respectively). Surgeries for soccer injuries accounted for the least proportion (*p* < 0.05), and surgeries for ball strike injuries were less than those for racket/equipment beating injuries (*p* = 0.046). The proportions of eye surgery and removal were significantly different among the specific causes (*p* < 0.001and *p* = 0.005, respectively) and were less for injuries caused by ball strikes than those caused by physical collisions (*p* < 0.001and *p* = 0.024. respectively).

### 3.7. Changes in VA and outcomes

**Figure 2:**
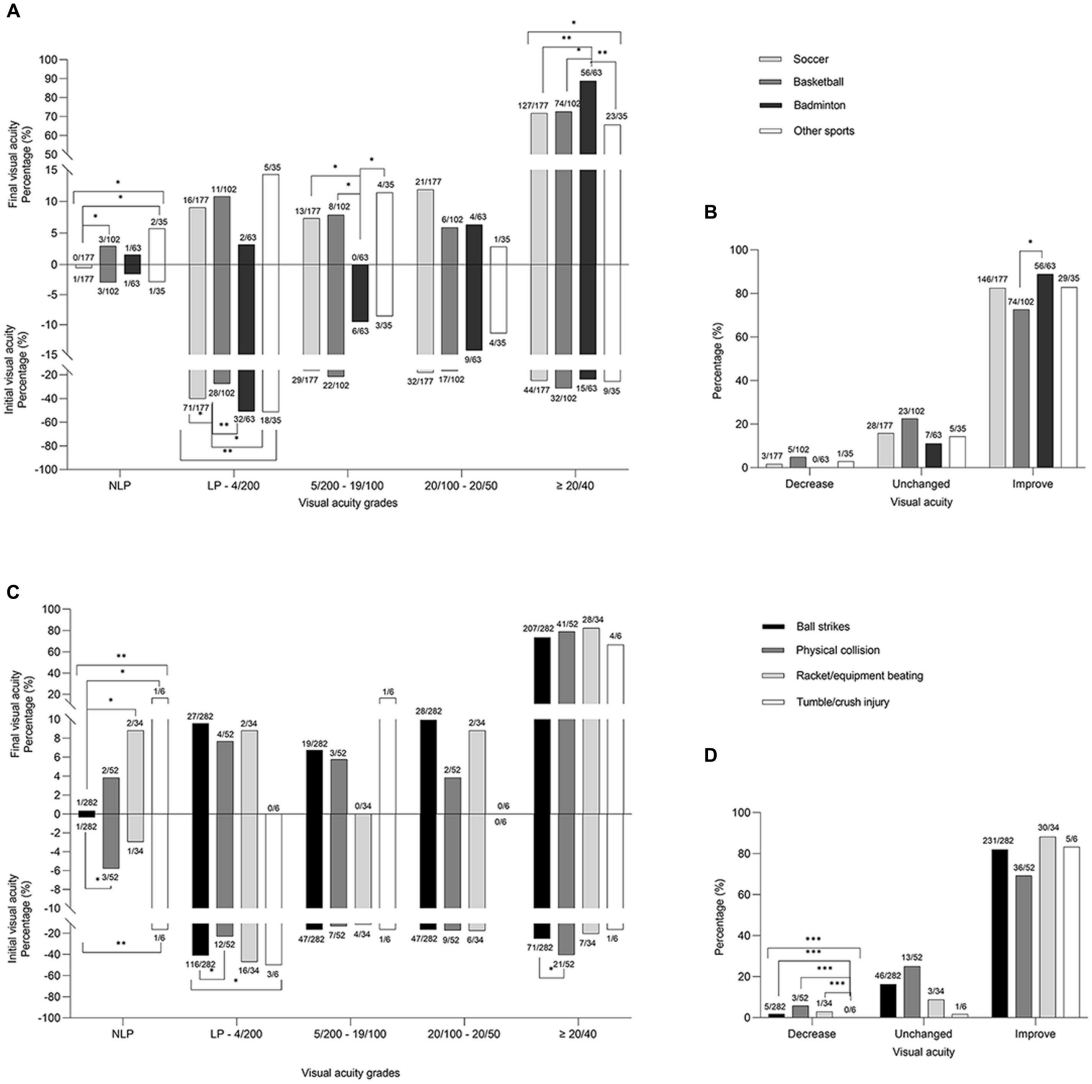
The comparision between the initial and final visual acuity **(A,C)** and the distributions of visual acuity changes **(B,D)** in percentage of different sports items **(A,B)** and specific causes **(C,D)**. Asterisks indicate significantly different composition proportions (* *p* < 0.05, ** *p* < 0.01, *** *p* < 0.001). LP, light perception; NLP, no light perception.

The presenting VA grades were significantly different from the final VA grades (*p* < 0.001). The presenting VA after sports-induced eye injury was most in light perception (LP)-4/200. A final low vision (≤4/200) was observed in 10.6% of the eyes (40/377; Table 4). This difference between the presenting and final VA existed in each type of sport (soccer, basketball, and badminton: all *p* < 0.001 and other sports: *p* = 0.002; Figure 2A) and in each specific cause (*p* < 0.001), except tumble/crush injury (*p* = 0.167; Figure 2C).

**Table 4:** Presenting and final visual acuity of eyes injured in sports.*

VA grades	Presenting VA	Final VA	*p* value
Grade 1 20/40 or better	100 (26.5%)	280 (74.3%)	*p* < 0.001
Grade 2 20/100–20/50	62 (16.5%)	32 (8.5%)	
Grade 3 5/200–19/100	60 (15.9%)	25 (6.6%)	
Grade 4 LP-4/200	149 (39.5%)	34 (9.0%)	
Grade 5 NLP	6 (1.6%)	6 (1.6%)	

VA, visual acuity; LP, light perception; NLP, no light perception. *377 eyes; the final visual acuity of NLP including the 3 removed eyes.

The presenting VA grades were not significantly different among the sports (*p* = 0.153) and among specific causes (*p* = 0.06), but the final VA differed among the sports (*p* = 0.017) and among specific causes (*p* = 0.035). The presenting VA in LP-4/200 grade was significantly different among sports (*p* = 0.009) and was highest in badminton (50.8%) and other sports (51.4%), followed by soccer (40.1%) and basketball (27.5%). Regarding the final VA, the proportions of no light perception (NLP; *p* = 0.019) and ≥ 20/40 VA (*p* = 0.028) differed among the sports items. Badminton had the highest proportion of ≥20/40 VA in the final VA result (88.9%, *p* < 0.05; Figure 2A).

The proportions of presenting VA in NLP and LP-4/200 grades varied among the different causes (*p* = 0.002 and *p* = 0.048, respectively). Ball strikes caused a lower percentage of NLP and a higher percentage of LP-4/200 than that caused by physical collision (0.4 vs. 5.8%, *p* = 0.012 and 41.1 vs. 23.1%, *p* = 0.019, respectively). Regarding the final VA, the NLP grades were significantly different among the specific causes (*p* = 0.002). Ball strikes had a lower proportion of NLP VA in the final VA measurement (0.4%) than that had by racket/equipment beating (5.9%; *p* = 0.032) and tumble/crush injury (16.7%; *p* = 0.041; Figure 2C), respectively.

At discharge or follow-up, VA improved in 80.9% (305/377), did not change in 16.7% (63/377), and decreased in 2.4% (9/377) of the eyes. However, the overall change in VA among the sports and the specific causes were not statistically significant (*p* = 0.151 and *p* = 0.170, respectively). Improved VA ratio was higher in badminton (88.9%) than that in basketball (72.6%; *p* = 0.013; Figure 2B). Deteriorated VA ratio varied significantly among the different causes (*p* < 0.001) and was less in tumble/crush injury than in other causes (*p* < 0.001; Figure 2D). Final VA was positively correlated with presenting VA (spearman coefficient 0.421, *p* < 0.001).

## 4. Discussion

### 4.1. Epidemiological characteristics

Sports-related eye injuries mainly affect young people in their 20s (14–19), which is similar to the age distribution in this study. In other rare instances, the incidence has been observed to be higher in teenagers (3, 20), late vicenarians (21) or quadragenarians (11, 22) for particular groups or sports. Men accounted for an overwhelming majority (78.9%–88.3%) (14–16, 19, 21, 22) and accounted for 93.9% participants in this study, probably because of their stronger built and speed and their increased participation in relatively high-risk sports compared with that observed in women. The extremely high percentage in our study compared with previous studies may be due to differences in periods, region, sample, and the types of sports included in the study.

### 4.2. Main causative sports and specific causes of sport injuries

The primary causes of sports injury vary among different countries (3, 8, 10, 14–16, 20–25), such as prominence of basketball in America (38.6%) (14, 19), badminton in Malaysia (66.6%) (23), floorball in Finland (32%) (26), hockey in Canada (44.19%) (24), cycling in western Australian (22%) (20), and baseball in Korea (30.5%) (21). Soccer, the most popular sport in the world, is the primary (18.2–32.5%) (15, 16, 22, 25) or important (3, 14, 20, 21) cause of eye injuries in many countries. Basketball (3.8–22.6%) (15, 16, 18, 21, 22) and badminton (1.3–14.6%) (15, 16, 21, 22, 25) are also widely reported as common causal sports. Soccer (46.8%), basketball (27.1%), and badminton (16.7%) were the most common causal sports for eye injuries in this study, with higher proportions than those reported in the previous literature. A survey of Chinese college students showed that these three were also the favorite sports among men (basketball, 54.1%; soccer, 32.2%) or women (badminton, 26.9%) students (10), which supports our findings.

Ball strikes (74.7%), physical collision (13.8%), and racket/equipment strikes (9.0%) were the common specific causes of eye injuries in this study. According to most literature, ball strike is the most common cause of eye injuries (42.4%–95.2%) (3, 9, 15, 16, 22, 27). Physical collision generally accounts for 12%–28.2% (3, 9, 16, 20, 25, 27), and even as high as 47.3%–64.5% in middle school and college students (28), respectively, predominantly in basketball games (15). Our study also found a similar observation (42.16%) reflecting a higher frequency and intensity of the collision among the players due to the fast speed and relatively limited space for movement in the basketball court compared with soccer; racket/equipment beating varied from 5.8% to 42.5% (3, 16, 20, 25) owing to the different proportions of racket/stick sports included in different studies.

### 4.3. Mechanisms of eye injuries in sports

Mechanical forces from sports activities can lead to closed or open globe injuries and can involve ocular appendages such as the eyelids, orbits, and optic nerve. The condition of sports-induced injuries is closely related to the characteristics of the objects that collided with the eye, such as its properties, contact area relative to the orbital rim diameter, volume and depth of entry into the orbit depth, maximum force and velocity, and hardness or elasticity (8, 29).

#### 4.3.1. Blunt or sharp objects

Collisions of blunt objects, such as balls, rackets, sticks, paddles, fishing pendant with heavy weights, and body parts, exert compressive forces on the eyeballs whose expansion perpendicular to the direction of impact, which has been proposed as the major cause of contusion (8). This explanation is consistent with the pattern of most of the trauma cases in this study. When a blunt external force is quickly applied to the eye and intraocular pressure is greatly elevated exceeding the capacity of the globe wall [computational models of blunt eye impacts reported the following threshold values: stresses in the corneoscleral shell >23 MPa and local dynamic pressures >2.1 MPa (29)], the eyeball ruptures (8). The rupture occurs mostly at the location of peak stresses, such as the apex of the cornea, the limbus, and the equator of the globe (30) or occurs at the weakness in ocular wall caused by previous surgery or trauma. In this study, one case of rupture caused by a hit from a shuttlecock had a history of penetrating keratoplasty.

Most small, sharp, or high-speed projectiles, such as fishhooks, shattered eyewear lenses, and shoes nails, can cut and even penetrate the eyeball wall, which has the highest ratio of lamellar laceration or even penetrating injuries. In this study, five out of six penetrating injuries were caused by broken lenses worn by the participants during sports activities. The possible conversion of a blunt eye trauma into a globe penetration injury or even an intraocular foreign body due to a broken eyewear and the subsequent occurrence of a permanent visual impairment cannot be ignored.

#### 4.3.2. Contact area

Larger diameter objects (> 5 cm in diameter), such as soccer ball, basketball, and most parts of the physical body, deliver a limited force into the orbit, because most of their energy can be dispersed across the surrounding orbital bones and face (8, 30). Conversely, a blunt object smaller than the orbital opening causes anteroposterior compression and dilation of the middle part of the globe, transmitting a great force to the internal ocular structure. Similarly, the fingers, for example, are more likely to cause penetrating trauma during a basketball game (7, 30).

#### 4.3.3. The extent of orbital invasion

The larger the volume and/or depth of the blunt object invading the orbit, the more severe the eyeball will be crushed or damaged (8). Blunt objects smaller than the orbital opening (sticks, fingers, fishing pendent, table tennis ball, etc.) can transmit greater forces to the globe than that transmitted by ones that are larger than the bony opening (soccer, basketball, tennis ball, the elbow, the fist, some rackets, etc.) can do.

#### 4.3.4. Peak force and velocity

The faster the speed and greater the mass of the injured object, the greater the kinetic energy, and the shorter the time to reach the peak force value, the stronger the impact and damage to the eyeball. For example, OGI developed more likely from a standard major league baseball at 23.2 m/s (55 mph) than a kicked soccer ball (31). Racket sports, such as badminton, table tennis, tennis, squash (8), which are popular in China, are a common cause of serious eye injuries. For badminton and tennis, the small, density and high-speed shuttlecock or tennis ball, respectively, from an opponent or a doubles partner is a reason for the high risk of eye injuries (8, 11). Although the table tennis ball is small and light, its speed can be very high during a competition because of the small table and the close distance between the players.

#### 4.3.5. Hardness or elasticity

The soccer ball, relatively slow and soft compared to other balls, can deform and mold on impacting the contour of the orbital aperture (32), and remains in contact with the orbit for approximately 10 ms, 2.5 to 10 times longer than other balls (8). The impact can generate transient pockets of high and negative pressure (a suction effect as it withdraws) with a peak of 66.6 kPa near the impact point, and the impact subsequently propagates linear pressure wave, exerting compressive and tractional forces on the retina, especially localized to the posterior pole or superior temporal quadrant (8, 33). The suction power can distort the globe and tear the internal ocular structures in the anterior (such as tearing of sphincter pupillae or peripheral edge of the iris, recession of the anterior chamber angle, and ciliary body detachment from the scleral spur) and posterior segments of the eye (such as acute posterior vitreous detachment, dialysis of the ora serrata, retinal tears, macular holes, retinal or choroidal laceration, and optic papilla avulsion) (34). Furthermore, clinically, the insignificant coup and contrecoup mechanical injury can also generate commotio retinae (35). However, hard balls (field hockey, baseball, softball, polo ball, and golf ball) rebound in a fifth to a tenth of the time of the soccer ball, whose hardness quickly transmits energy or deeply penetrates into the orbit, which more likely cause eyeball rupture (8).

### 4.4. Peculiarity of sports-induced mechanical eye injuries

Blunt force injuries (contusion and rupture) predominates in sports-related ocular trauma. This was observed in the present study (361/377, 95.8%) as well as most previous related reports (3, 7, 11, 16, 20, 27), which is different from the pattern of some other causes of eye injuries (2, 5).

CGI, especially contusion, accounts for the majority of the eye injuries in all sports groups (95.1%) and specific causes groups (349/377, 92.6%) in this study, which is similar to or even more than other sports-related reports (contusion, 29.1%–77%) (3, 7, 9, 19, 21, 28). The difference in data may be due to the fact that the study sample comprised hospitalized patients with more severe eye injuries, and some sports such as hockey, baseball and tennis, which are more aggressive, were excluded.

In this study, the occurrence of OGI (4.9%; 12 eyes of rupture by blunt injury and 6 eyes penetrated by a sharp object) was relatively higher in basketball (8.3%) and other sports (12.9%) than in soccer (1.1%), and in physical collision (12.8%) and racket/equipment beating (11.8%) than in ball strikes (2.1%). OGI generally constitute a minority of injuries in sports (0.7%–7%) (3, 19–21) but constitute an exceptionally high proportion in golf (36). In this study or other literature (29), soccer was not identified as a sport that is commonly associated with OGI, excepting the related secondary injuries that are caused by inappropriate equipment (21), such as broken eye-wears and shoe snails, which were responsible for all the cases of penetration in this study. Globe rupture depends on the area-normalized kinetic energy caused by the contact object, which synthesizes relative in size to the area of the eye, mass, velocity and kinetic energy (26). Basketball sports which involve a fiercer confrontation compared with that in soccer may be the reason why basketball is more prone to causing area-normalized kinetic energy and eyeball rupture. In this study, one case of eyeball rupture was caused by a baseball. However, such small, hard, and fast ball or rod striking sports are not popular in China and the sample size was too small, accounting for the low proportion of eyeball rupture injury in this study. Regarding characteristics of velocity, stiffness and geometric difference, physical collision and racket/equipment beating in sports is obviously more likely to generate higher area-normalized kinetic energy than that generated by large balls.

The affected zone of injured eyes differed by sports or specific causes. According to a computer simulation study, soccer ball and basketball, relatively slow, soft and large balls, exert a primary linear force (pushing) and subsequent suction power (pulling) mainly to zone III (posterior segment) of the eyes (33). The bigger weight (630 g) and the faster and more hush physical contact of the basketball (37) contribute to a higher impact force to zone I than that observed with the soccer ball and shuttlecock. However, the large and smooth interface of the basketball prevents it from further squeezing and deforming the eyeball, which less often leads to damage in zone II compared with that caused by the shuttlecock in badminton. The small and dense shuttlecock that can produce a speed of over 200 kph (38) and racket/equipment are made of hard materials with much higher kinetic energy than that transferred to the ball and even shapes that are smaller than the eye sockets (power twist, stick, fishing pendant, etc.), which can allow direct globe compression deformation, mainly endangering zone II.

### 4.5. Ocular clinical manifestations

Hyphema (52.8%) was the most frequent clinical feature in ocular sports injury as reported by other literatures (28.9%–87.5%) (3, 11, 15, 16, 21, 22, 25, 38), and was particularly highest in badminton sport (85.7%), similar to that reported in a previous literature (94%) (38). Hyphema is a direct trauma that causes the shearing of forces on the blood vessels of the iris (39) located in zone II of the eyeball, which is consistent with the evidence that badminton is prone to affect zone II, as indicated above. Similarly, secondary glaucoma caused by hyphema, anterior chamber angle recession or forward of iris-lens septum mainly involve tissues in zone II. The proportion of such injuries caused by badminton is significantly higher than caused by other sports. Retinal breaks more frequently caused by soccer and basketball, fully corroborates the previous hypothesis that the vitreous body tracts on the retina (both belonging to zone III) due to the large ball rebound force. Any damage to the vascularization network of the uvea or retinal vessels can hemorrhage into the vitreous cavity.

### 4.6. Impact on visual acuity in sports participants

The initial low vision (≤4/200) accounted for as high as 41.11% (155/377) of the inpatients, who had severe ocular symptoms in this study, which was more than that (11%) reported among outpatients in the literature (15). In this study, sports-related eye injuries were mainly CGI, especially contusion, so the proportion of final vision ≥20/40 (74.3%) was similar to that reported in the literature for similar condition (68.5%) (40), but extremely higher than that reported in the literature on OGI (23.9%) (41).

The results of this study suggest that the VA prognosis in ocular sports injuries is closely related to the causative sports and/or the specific causes. For example, badminton caused the most MEI in zone II, where tissue damage mostly led to cloudy optical media in the anterior segment, such as hyphema and lens abnormality, resulting in poor initial VA following injury. However, the tissue damages in zone II were relatively resolvable by surgery or conservative treatment. Therefore, in this study, the ratio of improved VA was higher, and the ratio of a final VA of ≥20/40 was also the highest in the badminton sport group. Ball strikes caused more CGI and less OGI (destruction of the integrity of the eyeball and more serious damage to the intraocular tissues), which implied less surgeries; therefore, the percentage of NLP, VA, and enucleation was low.

### 4.7. Emphasis on effective avoidance and eye protection

Although all the main sports involved in this study, such as soccer, basketball, badminton are moderate-risk sports (7, 8), the permanent visual impairment of patients who experience related eye trauma is also very prominent and cannot be ignored. A systematic review indicated that the median percentage of eye injuries in racket sports due to no eyewear was found to be as high as 93% and to be especially noted was that wearing prescription lenses, contact lenses, and industrial eyewear in sports led to more severe eye injuries (42). Only protective equipment, such as polycarbonate lenses that adheres to the updated American Society for Testing and Materials (42), rather than regular lenses that are one-twentieth as strong (43) and can cause indirect eyeball penetration injuries, is necessary and effective in sports to reduce sports-related ocular trauma, especially in collision or contact sports (7). Eye injuries declined steeply following the introduction of visors, which was mandated for use during the national hockey league in 2013 (44). The establishment of safer surroundings and sports rules for participants also requires more attention. About 12.6% of all athletes are reported to use protective equipment for their eyes, reflecting the lack of eye protection awareness (9). Based on the developments in scientific protective eye-wears or masks, it is important to strengthen the safety awareness regarding protection against sports-related injuries.

### 4.8. Limitations

Although this study investigated multiple sports and drew polycentric data across a 5-year period, providing a broader overview of sports-related eye injuries in China, the limitations of observational study cannot be ignored. First, a selection bias might have occurred due to the limitations of sampling hospitals and inpatients, which excluded outpatients and emergency patients. Second, the sample size was relatively small. Third, there was a potential limitation in the integrity and validity of data. Fourth, not all evaluated cases had long-term follow-up data, and the long-dated changes and treatment outcomes of some injured eyes were unknown. Fifth, due to the lack of accurate records of relative afferent pupillary defect in many medical records, ocular trauma scores (45) cannot be used to evaluate and compare vision outcomes impaired by different sports or injury causes. Lastly, this study mainly analyzed popular sports in China, but lacks data on popular sports in other countries such as baseball. However, the detailed hospital records used in this study provided relatively comprehensive and accurate information on ocular tissue damage, treatments, and visual function.

## 5. Conclusion

This is the first report on sports-related eye injuries and the overall situation among the general population in China, based on multiple centers spanning 5 years. The clinical characteristics of eye injuries by various causal sports and specific causes are different. In soccer, ball strikes caused more injures to the posterior segment of the eyeball. OGI caused by physical collision of a player with basketball and racket/equipment beating should not be ignored. Badminton causes more Zone II injuries, and the visual prognosis is relatively better compared with that of other zone injuries. The VA prognosis was closely related to ocular tissue injuries and initial VA following a sports injury. The high proportion of final low vision and permanent visual impairment highlight that effective eye protection among sports participants is imperative and important.

## Data availability statement

The original contributions presented in the study are not included in the article/supplementary material, further inquiries can be directed to the corresponding author.

## Ethics statement

The studies involving humans were approved by Institutional Review Board of Chinese PLA General Hospital (S2002-074-01). The studies were conducted in accordance with the local legislation and institutional requirements. Written informed consent for participation in this study was provided by the participants' legal guardians/next of kin.

## Author contributions

YZ designed the study. QY, JY, QW, ZZ, and HZ collected and analyzed the data. YZ, HJ, and XK drafted the initial manuscript. YZ revised the article critically. XK, and HJ reviewed and edited the article. All authors contributed to the article and approved the submitted version.

## Abbreviations

VA: visual acuity
MEI: mechanical eye injuries
OGI: open globe injury
CGI: close globe injury
LP: light perception
